# Dexmedetomidine as a Short-Use Analgesia for the Immature Nervous System

**DOI:** 10.3390/ijms25126385

**Published:** 2024-06-09

**Authors:** Anatoliy Logashkin, Valentina Silaeva, Arsen Mamleev, Viktoria Shumkova, Violetta Sitdikova, Yaroslavna Popova, Dmitrii Suchkov, Marat Minlebaev

**Affiliations:** 1 Laboratory of New Engineering Solutions for Modern Laboratory Research, Kazan Federal University, Kazan 420008, Russia; anatolijlogaskin@gmail.com (A.L.);; 2 Institut de Neurobiologie de la Méditerranée (INMED U1249), Aix-Marseille University, 13273 Marseille, France

**Keywords:** dexmedetomidine, neonatal rat, electrophysiology, neuronal activity, pain, sleep, cortex, hippocampus

## Abstract

Pain management in neonates continues to be a challenge. Diverse therapies are available that cause loss of pain sensitivity. However, because of side effects, the search for better options remains open. Dexmedetomidine is a promising drug; it has shown high efficacy with a good safety profile in sedation and analgesia in the immature nervous system. Though dexmedetomidine is already in use for pain control in neonates (including premature neonates) and infants as an adjunct to other anesthetics, the question remains whether it affects the neuronal activity patterning that is critical for development of the immature nervous system. In this study, using the neonatal rat as a model, the pharmacodynamic effects of dexmedetomidine on the nervous and cardiorespiratory systems were studied. Our results showed that dexmedetomidine has pronounced analgesic effects in the neonatal rat pups, and also weakly modified both the immature network patterns of cortical and hippocampal activity and the physiology of sleep cycles. Though the respiration and heart rates were slightly reduced after dexmedetomidine administration, it might be considered as the preferential independent short-term therapy for pain management in the immature and developing brain.

## 1. Introduction

The myth of pain perception immaturity in neonates was rejected only 30 years ago [1,2]. Characteristic behaviors associated with pain, as well as endocrine and physiological stress responses in addition to non-physiological stimulation of the somatosensory system, have been demonstrated in neonates [2,3,4]. Insufficient analgesia during and following painful manipulations with neonates results in increased mortality and morbidity. The delayed effects of inadequate analgesia have also been shown [5,6,7]. Effective pain management remains critically important because of ethical reasons, and for short- and long-term outcome perspectives [8]. Exposure to pain due to the lack of or inadequate anesthesia in neonatal infants (especially those born very preterm) can cause negative short- and long-term effects. Decreased physiological stability, such as increased heart rate and decreased respiration rates, is often observed in response to acutely painful procedures [9,10]. The long-term effects include altered pain thresholds, neurocognitive development (including alterations in brain structure), behaviour, and cognitive ability, and is reported to be present in school-age children [11,12,13,14,15,16]. But pain management is challenging in neonates (especially immature ones) as we know little about the short- and long-term harm of anesthetics and analgesics [17]. From this point of view, dexmedetomidine (DEX) could be the drug of preference, as it is well tolerated in preterm infants [18]. Dexmedetomidine is a highly selective α2-agonist that possesses sedative, analgesic, opioid-sparing, and anxiolytic properties [18]. Though DEX is increasingly commonly used in anesthetic practice for various purposes, including sedation, premedication, and as an adjunct to other anesthetics, it is not yet licensed for use in pediatrics as an independent anesthetic (see [19], for review). Being off-label medication, it is administered as an adjunct to other anesthetics, most of which affect the functional interactions between the neuronal cells and thus modify neuronal network activity [20,21]. In the immature nervous system, most neuronal activity is sensory driven [22] and critically contributes to the formation of neocortical organization. Interference with the cellular mechanisms involved in immature neural activity generation, through the administration of volatile anesthetics for general anesthesia, results in neuronal apoptosis in the developing nervous system [23,24,25,26]. Modulation of immature neuronal activity by sensory deprivation during the late in utero or early postnatal period results in alterations to cortical map development in the sensory systems ([27,28,29,30,31]; see [32], for review). It is arguably the crucial supracellular mechanism of the development of the brain’s diverse functional organization, and modulation of it affects the development of brain structures. Immature patterns of neuronal activity are unique and organized in oscillatory bursts—early gamma oscillations and spindle-bursts, with the dominant frequencies lying in the gamma (30–80 Hz) and alpha/beta (8–30 Hz) frequency ranges, respectively [30,33,34]. In rodents, these activity patterns persist during the early postnatal period, and are observed during the second part of human gestation, suggesting these patterns are part of a common mechanism of sensory system development ([35]; see [36], for review). A unique immature activity pattern (early sharp waves, eSPWs) also characterizes hippocampal development. Being the first pattern of neuronal network activity in the hippocampus, eSPWs are thought to contribute to the development of hippocampal networks ([37,38]; see [39], for review). Therefore, the choice of an adequate mode of analgesia or anesthesia that does not cause significant changes in the activity of neuronal networks in the developing immature nervous system during its critical period, and at the same time provides adequate analgesic efficacy, is of paramount importance. Despite a wealth of clinical experience with dexmedetomidine, which demonstrates its effectiveness as a sedative–analgesic agent, the pharmacodynamic impact of dexmedetomidine on the patterns of immature neuronal activity remains largely unknown. In order to fill this gap, we compared the effects of dexmedetomidine with the effects of urethane, which is a widely used anesthetic in neuroscience experiments, with 1817 papers identified in PubMed (in a search for “urethane” AND “brain” from 2003 to 2023). Though the use of urethane as an anesthetic has diminished recently, the effects of urethane on cortical activity are well known. Moreover, comparative studies with different anesthetics showed that the functional connectivity pattern under urethane anesthesia is closer to that in the non-anesthetized animal [40,41]. Thus, in this study, using the neonatal rat as a model, we aimed to investigate the effects of dexmedetomidine as a potential analgesic and to consider its effect on immature patterns of cortical and hippocampal activity, comparing it with urethane, another well-known anesthetic.

## 2. Results

### 2.1. Modulation of Cortical and Hippocampal Activity by Dexmedetomidine and Urethane

**Figure 1:**
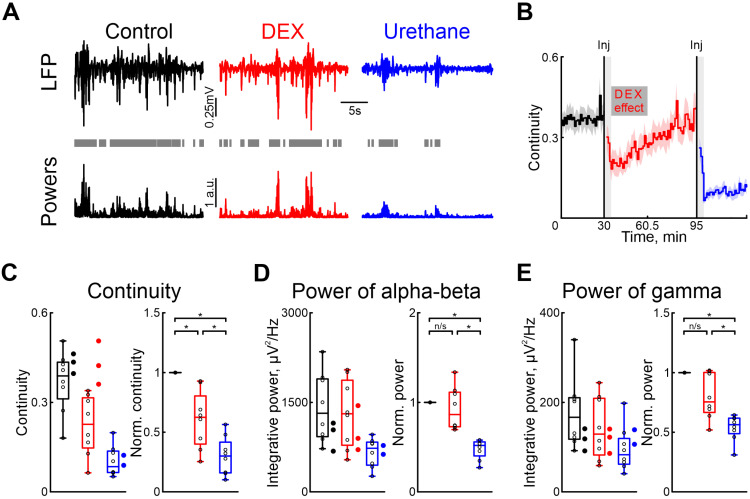
Effects of dexmedetomidine and urethane on the cortical activity. (**A**) Thirty-second episodes of cortical activity recorded in control, under dexmedetomidine, and under urethane conditions (black, red, and blue traces, respectively) are shown at the top. The integrative power of the alpha/beta and gamma frequency ranges for the segments shown above is displayed at the bottom. Gray rectangles mark detected activity. (**B**) The temporal profile of the continuity of cortical activity in control conditions, and under the influence of dexmedetomidine and urethane. The effect of urethane lasted beyond the duration of the experiment; therefore, only a thirty-minute period is shown. The vertical lines indicate the time of dexmedetomidine and urethane injections, respectively. Gray vertical rectangles of 5 min duration highlight the periods during which the animal calmed down following the injections. The horizontal rectangle represents the significant changes evoked by dexmedetomidine (DEX) injection. The stairs diagram illustrates the mean value of continuity of cortical activity (n = 8 P5–7 neonatal rat pups), and the shaded area indicates the jackknife deviation (black for control, red for DEX, and blue for urethane). (**C**–**E**) Group data for absolute and normalized relative to control values (left and right plots, respectively) of the continuity, integrative power in alpha/beta and gamma frequency of cortical activity ((**B**–**D**), respectively). The conditions are color coded: black—control, red—dexmedetomidine, blue—urethane. The filled circles represent the sham injection (black), low-concentration DEX (red), and urethane anesthesia without prior injections of DEX (blue). The middle line reflects the median, the upper and lower contours of the “box” correspond to the lower and upper quartiles, and the whiskers characterize the minimum and maximum values of the sample. The circles correspond to the results of individual experiments. (n/s—non significant, *—*p* < 0.0167 (Bonferroni correction)).

To characterize the effects of dexmedetomidine on cortical and hippocampal activity, we also used urethane as a comparison. To assess the effect of dexmedetomidine action on patterns of neuronal activity in the immature nervous system, we performed a series of recordings of extracellular potentials in the barrel cortex of newborn rats (Figure 1A), followed by analysis of cortical oscillatory activity in the alpha/beta (8–30 Hz) and gamma (30–70 Hz) frequency ranges. Followed the recordings of cortical and hippocampal activity in control conditions, the sequential intraperitoneal injections of dexmedetomidine and urethane (in 1 or 2 h) were performed.

Our results showed that, in contrast to urethane, DEX affected the immature patterns of cortical activity less. Continuity of cortical oscillations was significantly higher under dexmedetomidine conditions than after urethane injection (Figure 1C, Table S2).

DEX administration did not significantly modify the power of spontaneous cortical activity, in both the alpha/beta and gamma frequency ranges, provoking a slight decrease (by 13.58%, Table S3, and by 24.52%, Table S4, respectively, n = 8 neonatal rat of P5–7, Figure 1D,E).

Injection of urethane diminished cortical activity by up to almost 50% for the alpha/beta and gamma frequency ranges (by 48.14%, Table S3, and by 43.64%, Table S4, respectively, n = 8 P5–7 neonatal rats, Figure 1D,E).

Though we used a dexmedetomidine concentration of 33 μg/kg [42], the clinical dose is much lower [43,44,45]. Therefore, we tested the effects of clinical concentrations of dexmedetomidine (1.5 μg/kg) on the cortical activity in neonatal rats. Our results showed no difference compared with control conditions for cortical activity continuity and changes in power of alpha/beta and gamma oscillations (n = 3 P5–7 neonatal rats, Tables S18–S20, Figure 1C–E). Similarly we observed no effect when a sham injection with saline solution was administered. Neither the duration nor the power of alpha and gamma oscillations were affected (n = 3 P5–7 neonatal rats, Figure 1C–E, see Tables S18–S20).

To investigate whether dexmedetomidine influences the effects of urethane on neuronal and motor activity, experiments were conducted without the prior administration of dexmedetomidine. Our results were consistent with those observed when urethane was administered following dexmedetomidine injection. In experiments where only urethane was injected, there was a decrease in cortical activity continuity as well as a reduction in integrative power across alpha/beta and gamma frequency ranges, similar to the outcomes observed following dexmedetomidine injections (Figure 1C–E, n = 2 P6–7 neonatal rats, Table S17), suggesting that the effects of urethane are not exacerbated by prior injection of dexmedetomidine.

**Figure 2:**
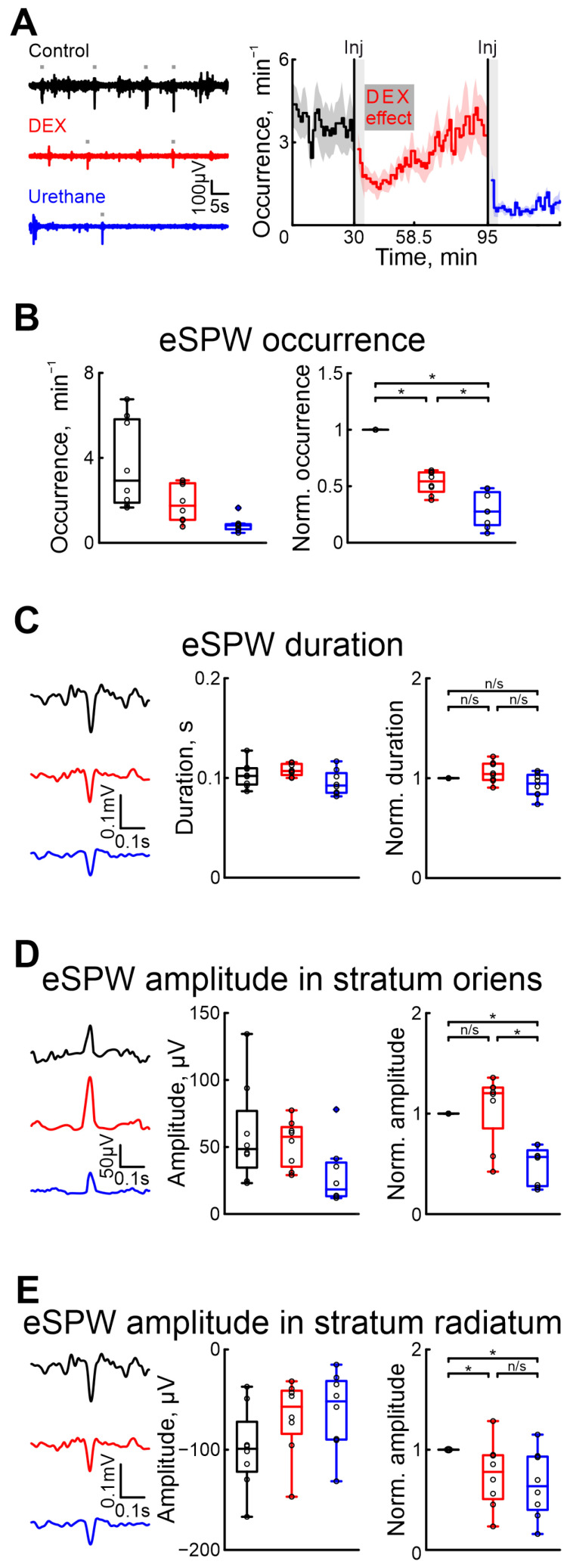
Effects of dexmedetomidine and urethane on the occurrence of early sharp waves and their amplitude–time characteristics. (**A**) Thirty-second episodes of hippocampal activity recorded in stratum radiatum in CA1 region in control, under DEX, and under urethane (black, red, and blue, respectively) are shown on the left. Gray rectangles indicates detected eSPWs. The temporal profile of eSPW occurrence in control conditions, and under the influence of dexmedetomidine and urethane are on the right. The stairs diagram illustrates the mean value of eSPW occurrence (n = 8 P5–7 neonatal rat pups), and the shaded area indicates the jackknife deviation (black for control, red for DEX, and blue for urethane). The effect of urethane persisted beyond the duration of the experiment; therefore, only a thirty-minute period is shown. The vertical lines indicate the time of dexmedetomidine and urethane injections. Gray rectangles of 5 min duration highlight the periods during which the animal calmed down following the injections. The horizontal rectangle represents the significant changes evoked by dexmedetomidine (DEX) injection. (**B**) Group data of absolute and normalized relative to control values (middle and right graphs, respectively) of eSPW occurrence. (**C**) Examples of spontaneous eSPWs in stratum radiatum in control, under DEX, and under urethane (black, red, and blue, respectively) are shown on the left. Group data of absolute and normalized relative to control values (middle and right graphs) of eSPW duration are shown on the right. (**D**) Examples of spontaneous eSPWs in stratum oriens in control, under DEX, and under urethane (black, red, and blue, respectively) are shown on the left. Group data of absolute and normalized relative to control values (middle and right graphs) of eSPW amplitude in stratum oriens are shown on the right. (**E**) Examples of spontaneous eSPWs in stratum radiatum in control, and under DEX and urethane (black, red, and blue, respectively) are shown on the left. Group data of absolute and normalized relative to control values (middle and right graphs) of eSPW amplitude in stratum radiatum are shown on the right. The middle line reflects the median, the upper and lower contours of the “box” correspond to the lower and upper quartiles, and the whiskers characterize the minimum and maximum values of the sample. The black circles correspond to the results of individual experiments. Black is control, red is DEX, and blue is urethane. (n/s—non significant, *—*p* < 0.0167 (Bonferroni correction)).

To evaluate the effect of DEX on hippocampal activity, we performed a series of recordings of extracellular potentials in the stratum oriens and radiatum of the hippocampus (Figure 2). The effects of DEX and urethane on spontaneous eSPWs were characterized. The results showed that, as in the cortex, DEX administration affected the eSPWs less than urethane.

As well as in the cortex, we assessed the onset and duration of dexmedetomidine’s effect to prevent overlap with urethane’s effect. Our results indicated that the effects of dexmedetomidine were observed within 4–5 min and lasted for approximatively 23.5 min (n = 8 P5–7 neonatal rats, Figure 2A, Table S1).

Five minutes after DEX injection, there was a 45.74% drop in the occurrence of eSPWs compared with control conditions, whereas urethane administration resulted in a more prominent drop (for 72.48%, n = 8 P5–7 neonatal rats, Figure 2B, Table S5). The duration of eSPWs was weakly modified by drug injection (increase of 4.14% for DEX, and decrease of 5.39% after urethane injection, Table S6, n = 8 P5–7 neonatal rats, Figure 2C).

In contrast to urethane, which significantly decreased the amplitude of eSPWs in both the strati oriens and radiatum (43.17% and 36.43%, respectively), DEX evoked a decreased eSPW amplitude in the stratum radiatum (of 22.19%), but not in the stratum oriens (non-significant increase of 20.33%, n = 8 P5–7 neonatal rats, Figure 2D,E, Tables S7 and S8). Results of sham or dexmedetomidine low concentration injections are shown in Tables S21–S24).

### 2.2. Pain Sensitivity, Sleep–Wake Cycles and Myorelaxation

**Figure 3:**
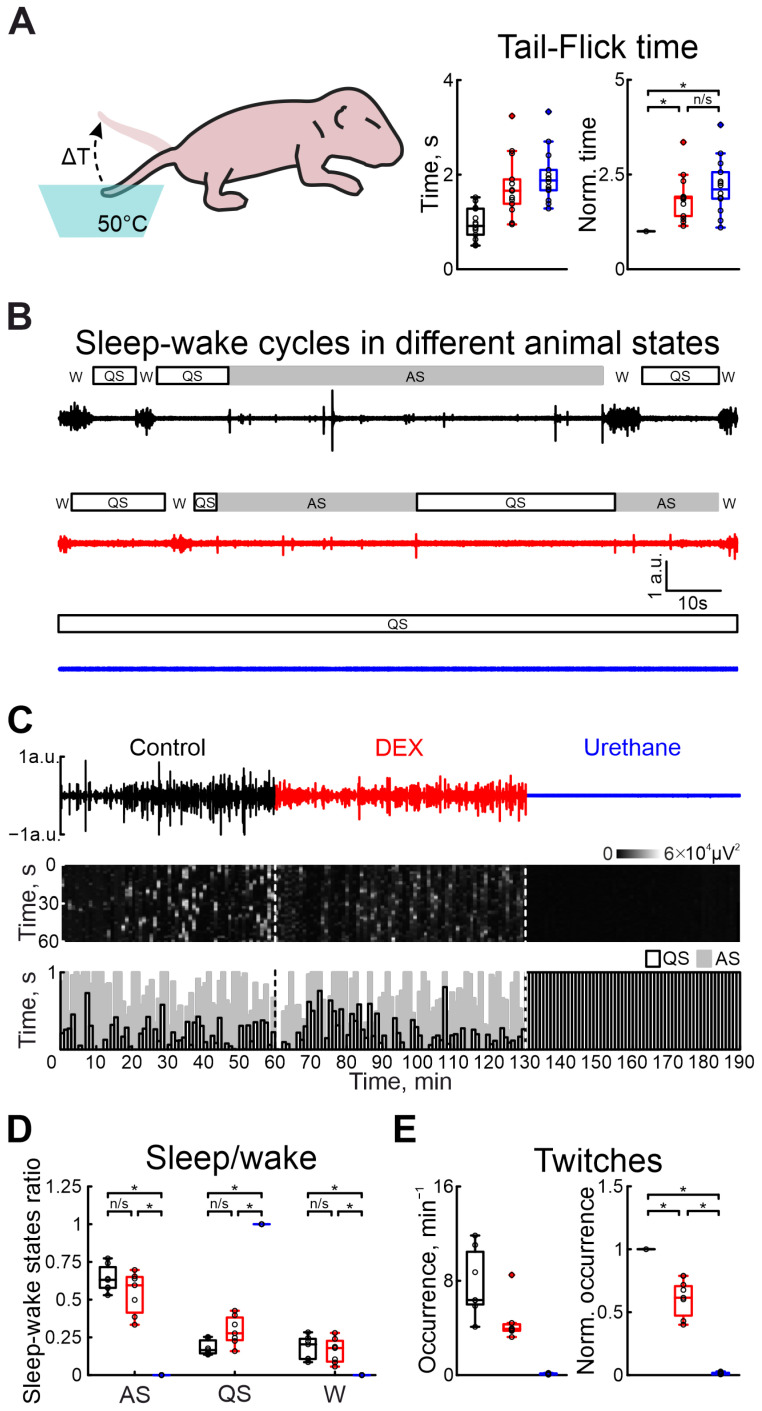
Effects of DEX and urethane on pain sensitivity, sleep–wake cycles, and myorelaxation. (**A**) The scheme of the experiment to evaluate pain sensitivity in newborn rats is shown on the left. Group data of absolute and normalized to control values (middle and right graphs, respectively) of time of animal’s tail flick in hot water in control, under DEX, and under urethane (black, red, and blue colors, respectively) are shown on the right. (**B**) Two-minute episodes of electromyogram (EMG) of cervical muscle activity in control, under DEX, and under urethane (black, red, and blue colors, respectively) with detected episodes of sleep–wake states of the neonatal rat. Quiet sleep (QS) episodes are rectangles with black outlines, active sleep (AS)—gray rectangles, and wakefulness (W)—empty spaces. (**C**) Three-hour duration cervical muscle electromyogram (EMG) recorded in control, under DEX, and under urethane (black, red, and blue colors, respectively). The results of cervical muscle activity detection are shown below. Moments of activity were detected in windows of 10 s each and are shown in white. The first white vertical dashed line is the DEX injection and the second is the urethane injection. The bottom stacked bar graph displays the animal’s condition. White columns with black outlines correspond to the state of quiet sleep, gray bars reflect active sleep, and empty space reflects the state of wakefulness. The first black vertical dashed line represents DEX injection, and the second line represents urethane injection. (**D**) Group data of normalized duration of animal states in relation to total recording time (QS—restful sleep, AS—active sleep, W—wakefulness) under different recording conditions (control—black, DEX—red, urethane—blue). (**E**) Group data of absolute and normalized relative to control values of occurrence of myoclonic twitches (left and right plots, respectively) in control, under DEX, and under urethane (black, red, and blue colors, respectively). The middle line reflects the median, the upper and lower contours of the “box” correspond to the lower and upper quartiles, and the whiskers characterize the minimum and maximum values of the sample. The black circles correspond to the results of individual experiments. Black is control, red is DEX, and blue is urethane. (n/s—non significant, *—*p* < 0.0167 (Bonferroni correction)).

To assess the analgesic effect of DEX in the immature nervous system, the tail-flick test in the neonatal rat pup was used (Figure 3A). Administration of DEX or urethane strongly increased the latency of the tail-flick time. While the tail-flick test in control conditions showed a latency of 0.92 s (n = 14 P5–7 neonatal rats), administration of DEX significantly increased it by 87.97% (Table S9). Results of sham or dexmedetomidine low concentration injections are shown in Table S25. Interestingly, the latency was comparable to the tail pullback time after urethane anesthesia (tail-flick time increased by 110.64%, Table S9), suggesting DEX has the same analgesic efficacy as an application of general anesthetic—urethane.

To characterize the vigilance state in the developing rat pup, a combination of electromyography and movement detection was used. Discrimination between awake, and active and quiet sleep states was based on the combination of the characteristic features: cervical muscle atonia, duration of movements, and frequency of their occurrence (see Section 4 for description). In contrast to urethane, DEX administration non-significantly modified sleep–wake cycling (Figure 3B–D). While quiet sleep states slightly increased (from 16.53% to 27.71% of total recording duration), the active sleep and awake state weakly decreased (from 63.10% to 59.50% and from 20.37% to 17.90%, n = 7 P5–7 neonatal rats, Tables S10–S12), relative to control values. Urethane injection resulted in the animal falling into a quiet sleep state (QS) associated with absence of movement activity until the end of the experiment (n = 7 P5–7 neonatal rats, Tables S10–S12). Results of sham or dexmedetomidine low concentration injections are shown in Tables S26–S28).

In parallel with the comparison of DEX and urethane actions on the central nervous system function, we compared their effects on motor activity. Firstly, we characterized the modulation of motor twitch occurrence. Both anesthetics significantly decreased the occurrence of myoclonic twitches. But the effect of DEX administration was less prominent than for urethane injection. While the DEX injection significantly decreased twitches by 38.54% (n = 7 P5–7 neonatal rats, Table S13), urethane application almost totally suppressed myoclonic activity, reducing it by 99.17% (from 6.36 movements/min to 0.07 movements/min, n = 7 P5–7 neonatal rats, Table S13, Figure 3E). Results of sham or dexmedetomidine low concentration injections are shown in Table S29).

### 2.3. Dexmedetomidine Pharmacodynamics

**Figure 4:**
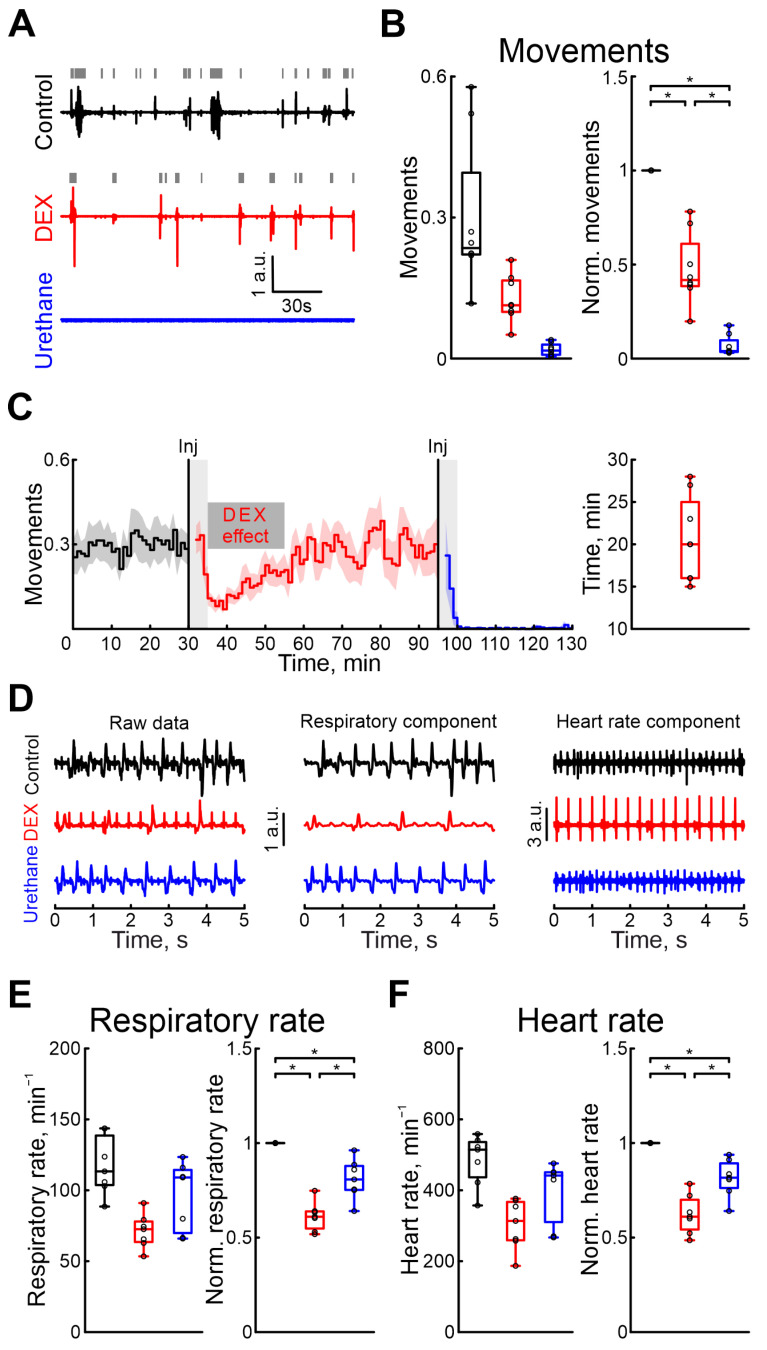
Modulation of motor activity, heart rate, and respiratory rate by dexmedetomidine and urethane. (**A**) Three-minute episodes of motor activity recorded in control, under DEX, and under urethane conditions (black, red, and blue colors, respectively). Gray rectangles indicate detected movements. (**B**) Group data of absolute and normalized to control values (left and right plots, respectively) of fractions of time of moving animal (black—control, red—DEX, blue—urethane). (**C**) Temporal profile of animal’s movements in control, and under dexmedetomidine and urethane conditions. The stairs diagram illustrates the mean value of animal’s movements (n = 8 P5–7 neonatal rat pups), and the shaded area indicates the jackknife deviation (black for control, red for DEX, and blue for urethane). The effect of urethane persisted beyond the duration of the experiment; therefore, only a thirty-minute period is shown. The first vertical line indicates the time of dexmedetomidine injection, while the second marks the time of urethane injection. Gray rectangles of 5 min duration highlight the periods during which the animal calmed down following the injections. The horizontal rectangle represents the significant changes evoked by dexmedetomidine (DEX) injection. (**D**) Piezo sensor recorded thorax movements with extracted respiration and heartbeats (left, middle and right columns, respectively) in control, under dexmedetomidine, and under urethane conditions (upper, middle, and bottom rows) are shown. Pharmacological conditions are also color coded (black—control, red—DEX, blue—urethane). (**E**,**F**) Group data of absolute and normalized relative to control values (left and right plots, respectively) of respiratory (**E**) and heart rates (**F**) are shown. Pharmacological conditions are also color coded (black—control, red—DEX, blue—urethane). The middle line reflects the median, the upper and lower contours of the “box” correspond to the lower and upper quartiles, and the whiskers characterize the minimum and maximum values of the sample. The black circles correspond to the results of individual experiments. Black is control, red is DEX, and blue is urethane. *—*p* < 0.0167 (Bonferroni correction)).

The effects of pharmacological agents were also compared using the rat pups’ motor activity, respiration, and heart rates. Firstly, we characterized the duration of DEX sedative effects. Our results showed that, 20 min (n = 8 P5–7 neonatal rats, Table S1) after the onset of recording, the motor activity was restored to the control values (Figure 4C). The time the animal moved was significantly reduced during the DEX action by 58.24% (Table S14), but urethane almost completely blocked any animal movements (movement activity decreases by 96.02%, 8 P5–7 neonatal rats, Table S14, Figure 4A,B. Results of sham or dexmedetomidine low concentration injections are shown in Table S30). DEX was shown to have effects on the cardiovascular and respiratory systems. While these effects were primarily observed with clinical dosages, we also tested them using high concentrations of DEX. DEX also significantly reduced the respiratory rate by 38.96% (Table S15. 7 P5–7 neonatal rats) and heart rate by 38.96% compared with control conditions (Table S16. 7 P5–7 neonatal rats, Figure 4D–F). The duration of DEX’s effect on respiratory rates lasted 26 min (4 P6–7 neonatal rats, Table S1), and on heart rate 23 min (4 P6–7 neonatal rats, Table S1). In contrast to dexmedetomidine, urethane anesthesia showed fewer prominent changes in these parameters: respiratory rate decreased by 19.25% (Table S15) and heart rate by 18.33% (Table S16. 7 P5–7 neonatal rats). Results of sham or dexmedetomidine low concentration injections are shown in Tables S31 and S32.

## 3. Discussion

Our results reveal that dexmedetomidine has prominent analgesic properties similar to those observed under general anesthesia evoked by urethane. However, unlike urethane, dexmedetomidine has a lesser impact on the immature patterns of neuronal activity in both the neocortex and hippocampus. The normal structure of sleep was maintained during dexmedetomidine application, but respiration and heart rates were decreased, which should be kept in mind.

The question of pain management in neonates, especially in immature ones, is extremely important and non-trivial. The balance between loss of pain and sensation, and the functioning of the immature central nervous system should be maintained. Neonatal pain was shown to have adverse effects, in both the short and long term. However, modulation of immature neuronal activity has a strong negative impact on nervous system development. Dexmedetomidine showed a strong analgesic effect, which was comparable with effects of general anesthesia evoked by urethane. The tail-flick test is a classical test to characterize pain sensitivity [46]. Our results demonstrated that the administration of dexmedetomidine significantly increased the time of tail removal from hot water; similar results were also shown in adult rats [47]. From an analgesic standpoint, our findings demonstrate for the first time that the effect of dexmedetomidine as an independent analgetic in the neonatal brain is in agreement with the pain-relieving effect of dexmedetomidine in adults, which has already been demonstrated in various models. Systemic administration of dexmedetomidine produces a dose-dependent state of sedation, in both humans and rats [48,49]. Using intracellular recordings from neurons of the locus coeruleus, activation of inwardly rectifying potassium channels was shown after dexmedetomidine exposure [50]. Unlike most other anesthetics that act predominantly via modulation of glutamatergic and/or GABAergic transmission, dexmedetomidine inhibits norepinephrine release in a downstream structure [51,52]. And that could be the crucial advantage of dexmedetomidine use as a short analgesia in the developing nervous system, as its analgesic effect is not linked to modulation of GABA and glutamate transmitter release. Recently, a number of studies rose safety concerns about the general anesthesia evoked by most pharmacological agents. Their interference with GABAergic or glutamatergic transmission provokes the neurodegeneration in the developing brain [23] (see [25], for review).

Interestingly, in contrast to urethane that suppressed neuronal network activity in both the cortex and hippocampus, dexmedetomidine strongly inhibited the occurrence of hippocampal eSPWs, while the continuity of cortical activity was preserved. Suppression of hippocampal activity could be explained by general sedation of the neonatal rat pup and thus decreased movement activity (including twitches). eSPWs are mainly bottom-up events involving inputs from the entorhinal cortex, which are activated during myoclonic movements (startles and twitches), and therefore the decrease in movement activity is followed by the drop in eSPWs (see [39], for review). However, cortical activity partially depends on sensory input. Sensory deprivation decreases the occurrence of immature oscillations; however, they still persist.These findings are consistent with other studies. Severing the sensory periphery or sensory pathways did not eliminate spindle bursts in the corresponding cortical representations but did reduce their incidence [22,31]. The critical period coincides with the expression of unique immature oscillations (spindle bursts and early gamma oscillations) [22,33]. Sharing the same dominant frequency ranges with the adult patterns of cortical activity (spindle burst with sleep spindle and early gamma oscillation with gamma oscillation), the immature patterns of cortical activity serve for other functions [31,53]. Surgical or pharmacological suppression of immature cortical activity results in alteration of cortical map development [27,54]. Therefore, preservation of the immature oscillatory activity is crucial for the development of cortical maps. Both the continuity and the power of immature cortical activity were preserved under dexmedetomidine. Our results also showed weak effects of dexmedetomidine on sleep and wake cycles in the developing brain. Using adult rodents, dexmedetomidine was shown to mimic natural sleep, and a normal physiological sleep and wake cycle was maintained [55,56,57]. Though the mechanism of dexmedetomidine analgesia has not yet been fully clarified, it is believed that its analgesic effect is primarily due to its action as a highly selective α2-adrenergic receptor agonist. This mechanism involves several pathways: (1) peripheral analgesic effect, produced by inhibiting the transmission of pain signals through Aδ and C fibers (similar to the mechanism suggested for clonidine); (2) central analgesic effect, via mediation of α2-adrenergic receptors in the locus coeruleus and the descending noradrenergic pathway of the spinal cord, inhibiting the release of substance P and other nociceptive peptides in the presynaptic membrane, thereby inhibiting the transmission of noxious stimuli in the spinal cord, which in turn terminates the signaling of pain; and (3) local analgesic effect, modulation of hyperalgesia by stimulating the α2 receptor [50,58,59] (see [52,60], for review).

While urethane primarily affected the central nervous system, dexmedetomidine also impacted the cardiovascular and respiratory systems. We observed an almost twofold decrease in heart and respiratory rates in our experiments. In control conditions, the neonatal rat heartbeat was shown to vary around 400 beats per minute (Table S16), being in agreement with previously shown results by other authors [61], and administration of dexmedetomidine significantly decreased the heart rate to 313.45 BPM (median value). Though it is lower than in control conditions, the marked decrease in heart rate has been shown to be compensation for peripheral vasoconstriction and thereby hypertension. We did not measure blood pressure during our experiments, but dexmedetomidine injection (which produced a high plasma concentration) resulted in an increase in blood pressure combined with a marked decrease in heart rate [48]. This effect is thought to originate from α2-receptor activation in the vascular smooth muscles. However, with a decrease in dexmedetomidine concentration in plasma, the vasoconstriction attenuates as dexmedetomidine also activates α2-receptors in the vascular endothelial cells, which results in vasodilation and hypotension. Dexmedetomidine also evoked a significant drop in the respiration rate (from 113.33 to 72.55, Table S15), which was also lower than the physiological ranges at this age. Our results are generally in agreement with those shown by other authors [62]. It was demonstrated that dexmedetomidine administration evoked hypopnea, but in association with modification of the respiratory pattern. The respiratory pattern became slower but deeper. Surprisingly, in spite of a decreased breath rate, in adult patients with chronic obstructive lung disease, dexmedetomidine infusion improves oxygenation [63,64]. Various mechanisms of the favorable respiratory effects of dexmedetomidine have been proposed (bronchodilation [65], increase in lung perfusion [66], and nitric oxide [67]). Moreover, the dexmedetomidine concentration used in our experiments was much higher than used for humans. We used the concentration 33 μg/kg, while the clinical dosage of dexmedetomidine for short-term sedation in adult humans in ICU is much lower (up to 1.5 μg/kg in 24 h) [44,45]. Therefore, the effects of dexmedetomidine on the cardiovascular and respiratory systems could be less obvious in neonates using the clinical dosage. In conclusion, based on the association of dexmedetomidine-induced analgesia with its weak effects on the structure of immature neuronal activity patterns in both the cortex and hippocampus, we suggest that dexmedetomidine could be an effective and safe alternative for pain treatment and sedation in short-term procedures associated with pain in the immature brain.

## 4. Materials and Methods

### 4.1. Ethical Approval

The study was conducted in accordance with the Declaration of Helsinki and approved by the Ethics Committee of Kazan Federal University (#24/22.09.2020). Efforts were made to minimize the numbers of animals used in the study. Every litter with its mother were kept in a single cage in a temperature-controlled room at 22 ± 3 °C under a 12/12 h light/dark cycle (light on at 7 a.m.). Standard food pellets and tap water were available ad libitum.

### 4.2. Surgery

Wistar rats of both sexes from postnatal days [P] 5–7 were used (P0 was the day of birth, n = 26). Surgery was performed under isoflurane (Baxter, Guayama, PR, USA)anesthesia (5% for induction and 1–2% during surgery). The rat skull was cleaned of skin and periosteum and covered with an acrylic (Meliodent RR, Hanau, Germany), except for a 5 × 5 mm window above the hippocampus and the somatosensory system (the coordinates were calculated using an atlas of the neonatal rat brain). We used the local application of bupivacaine during the surgery to provide local analgesia in addition to general anesthesia produced by isoflurane. A superfusion chamber (patent application no. 2020140611 of 09/12/2020, [68]) was then fixed to the animal’s head so that the cement-free area faced into the chamber. To have access to the brain structures of interest, holes of 0.2–0.3 mm diameter were drilled in the skull above the somatosensory cortex and hippocampus for installation of extracellular electrodes. After the surgery, the rats were fixed in the setup, warmed, surrounded by a cotton nest, heated via a thermal pad (35–37 °C), and left for an hour to recover from anesthesia before electrode implantation and start of experiment.

### 4.3. Pain Sensitivity Test

To characterize the pain response, the classic test—tail-flick test—used in basic pain research and to measure the effectiveness of analgesics was used [46,47,69]. Briefly, the tip of the tail was lowered into a water bath with a temperature of 50 °C. Latency to tail withdrawal was recorded as the “pain” characterizing parameter, with a 15 s maximum time period. After three habituation tests, the latency to withdrawal was determined from the average of three consecutive measurements for each experimental condition.

### 4.4. Cortical and Hippocampal Activity Recordings

Cortical and hippocampal activity was recorded using custom-made three-channel electrodes made from 50 μm diameter twisted nichrome wires (California Fine Wire Co., Grover Beach, CA, USA). The tips of the wires were cut at different lengths, with an increment of 200 μm, to track neuronal activity simultaneously at different depths. The thalamorecipient layer (layer 4, granular layer) used for the cortical activity recordings was identified by two criteria: (i) the depth calculated using a neuroanatomical atlas and (ii) the most negative amplitude of the sensory evoked potential with an evoked potential onset delay corresponding to the age of the animal [53]. The hippocampal activity was characterized using simultaneous recordings from the oriens, and pyramidal and radiatum layers. The electrodes were positioned to record the pyramidal layer with the middle electrode (the pyramidal layer was considered as the layer with reversal of early sharp-wave events and intensive neuronal firing).

A chlorosilver wire was used as a ground electrode and was placed into the cerebellum. Recorded local field potential (LFP) was amplified and filtered (×10,000; 0.15 Hz to 10 kHz, respectively) using a Digital Lynx SX amplifier (Neuralynx, Bozeman, MT, USA) with a sampling rate of 40 kHz or an Open Ephys Acquisition Board amplifier (Open Ephys, Atlanta, GA, USA) with signal amplification, filtering, and sampling of ×400, 0.1 Hz to 7 kHz, 30 kHz, respectively. The recordings lasted for one hour (three recordings of 2 h durations were carried out with dexmedetomidine) for each of three conditions (control, dexmedetomidine, and urethane administrations). The agents were injected intraperitoneally. Data analysis was performed post hoc.

### 4.5. Preparation and Administration of the Substances

Dexmedetomidine (Orion Pharma, Espoo, Finland) and urethane (Sigma-Aldrich, Saint Louis, MO, USA) were used at concentrations of 33 μg/kg and 1 g/kg, respectively. To assess the effects of a clinical dose of dexmedetomidine, its concentration was decreased to 1.5 μg/kg. Urethane and dexmedetomidine were dissolved in saline to make the injectable volume 100 μL/10 g of animal weight (total volume of injection was 120–240 μL/pup depending on its weight). The injections of dexmedetomidine and urethane were performed sequentially with an interval of 60 or 120 min.

### 4.6. Animal State Detection

The electrical activity of nuchal muscle was recorded simultaneously with cortical activity for post hoc analysis of the animal state. For that, two electrodes (of 50 μm diameter) were installed into the cervical muscles and fixed using cyanoacrylate glue. Using piezosensors, the animal’s motor activity, respiration, and heartbeats were also recorded. A piezoelectric element under the forelimb was used to record motor activity of the animal. Breathing and heartbeat were monitored by a second piezosensor placed under the pup’s thorax. The signal registered by the electrodes was amplified and filtered using a Digital Lynx SX amplifier, Bozeman, MT, USA (×10,000; 0.15 Hz to 10 kHz) or an Open Ephys Acquisition Board amplifier (Atlanta, GA, USA) (×400; 0.1 Hz to 7 kHz).

### 4.7. Data Analysis

Data analysis was performed using custom written functions in Matlab2021a (Mathworks, Natick, MA, USA). Firstly, all experimental recordings were preprocessed by lowering the sampling rate of the original signal to 1000 Hz to reduce file size.

To characterize the continuity of cortical activity and drug-induced modulation of its spectral properties, firstly, periods of network neuronal activity were detected. For that, a continuous time–frequency transform using a Morlet wavelet (mother wavelet of 6) was applied to estimate the integrative power in the alpha, beta, and gamma frequency ranges at each millisecond. Then, the integrative power was summed by 250 ms bins over the entire recording. The 250 ms window was chosen as it corresponds to 2 periods of the lowest frequency in the alpha band. The expectation–maximization algorithm was used to fit the integrative power distribution. Time periods where the signal amplitude exceeded the sum of the mean value and 2.5 standard deviations (that corresponds to *p* = 0.006) from the Gaussian distribution closest to zero (corresponding to the noise component) were considered as periods of cortical activity. The continuity of neuronal activity was quantified as the ratio of time with suprathreshold cortical activity per minute. The signal’s power was estimated as a sum of squared values of the wavelet transform coefficients in the frequency band of interest.

To determine the duration of the drug’s effect, we analyzed a minute-by-minute continuity value. The period with a continuity value lower than the threshold was considered as a period of drug action. The threshold was considered as the difference of a mean and 2.5 standard deviations calculated over the control period of recordings (that corresponds to *p* less 0.01). The first and the last minutes with a continuity value lower than the threshold were considered as onset and termination of drug action, and were used to calculate the drug effect duration. All analyses were performed during the periods of drug action, calculated as described before. The onset times slightly varied between the animals (4–5 min), and therefore we considered the onset point at 5 min after injection. As dexmedetomidine action times varied for different types of analysis (see Table S1), to be conservative, we used the shortest period of its action as 20 min (starting from effect’s onset time). Urethane acts longer than the duration of the experiment, and therefore there are no duration of urethane action data.

Early sharp waves are a characteristic pattern of neuronal network activity in the developing hippocampus in vivo. eSPWs were detected in two steps. Firstly, the LFP was filtered using a Chebyshev bandpass type II filter (1–20 Hz). All events whose amplitude exceeded 3 standard deviations of the LFP fluctuations (calculated over the entire recording) were considered as eSPWs. All detected events were checked manually by the operator. An event was counted as an eSPW if several criteria were met: (i) reversal in the pyramidal layer of the CA1 region, and (ii) absence of lateral “shoulders”, suggesting that the event was part of an oscillation. The changes in eSPW amplitude, duration in both the stratum oriens and radiatum, as well as eSPW occurrence served to characterize the effects of the pharmacological agents.

#### 4.7.1. Motor Activity Recordings

Motor activity of the animals was analyzed using a signal received from a piezoelectric element placed on the front right limb. To exclude the influence of low-frequency artifacts on the automatic detection process, the original signal was passed through a Chebyshev highpass type II filter (>1 Hz). The expectation–maximization algorithm was used to fit the signal’s absolute amplitude distribution (calculated over the entire filtered trace using the 10 ms window). Time periods with signal amplitudes exceeding the sum of the mean value and 2.5 standard deviations from Gaussian distribution closest to zero (corresponding to the noise component) were considered as periods of motor activity.

The respiratory rate (RP) and heart rate (HR) of the animal were determined using the signal obtained from a piezoelectric element placed under the chest of the animal. Signal wavelet-based filtering (0.5–3 Hz and 3–12 Hz for estimation of RP and HR, respectively) was performed to exclude low-frequency oscillations and isolate individual components with respiratory movements and heartbeats. Then, the mean RP and HR during the period of drug action were calculated to characterize the pharmacological agents’ action.

#### 4.7.2. Electromyography

Recordings of the electrical activity of the cervical muscles were used to characterize the changes in sleep–wake cycles, sleep phases, and the occurrence of myoclonic twitches. Firstly, the original signal was passed through a Chebyshev highpass type II filter (>120 Hz). The expectation–maximization algorithm was used to fit the signal’s absolute amplitude distribution (calculated over the entire filtered trace using the 10 ms window). Time periods with signal amplitudes exceeding the sum of the mean value and 2.5 standard deviations from Gaussian distribution closest to zero (corresponding to the noise component) were considered as periods of cervical muscle activity. All detected events were checked manually by the operator. To distinguish periods of animal wakefulness, the following criteria were used: (i) presence of high-amplitude continuous activity (high muscle tone) of more than 1 s (conditional boundary accepted on the basis of average duration of myoclonic twitches [70]), (ii) movements on the piezoelectric element during the entire period of activity, and (iii) cervical muscle atonia preceding and following the event.

The rest of the time, outside these events, was considered as sleep. For further differentiation of sleep into phases, twitch detection was also performed. Differentiation of sleep phases was made according to the following criteria, previously described by [71]. Briefly, quiet sleep (QS) was considered as the time period from the end of the waking period to the first twitch or to the beginning of the next waking period if there were no twitches between them, and active sleep (AS) began from the first twitch and to the next waking period, provided the next twitch occurred no more than 30 s later within this period (if this condition was not met, this period was considered to be the first period of active sleep).

#### 4.7.3. Statistical Analysis

Because of high variability of the values under drug action, they were normalized relative to corresponding control values in the same rat pup. Reliability of changes was considered using Wilcoxon’s test (for normalized to control values), with a significance level of 0.05 (*—*p* < 0.05). Because multiple hypotheses were tested, the Bonferroni correction was used to compensate for the increased probability of null hypothesis rejection. The variations on temporal profiles plots are shown by a shaded area that corresponds to Jackknife deviation. Variance charts (boxplot) were used to describe pooled data results, with the center mark indicating the median, and the lower and upper edges of the box indicating the 25th and 75th percentiles, respectively. The degree of variance was indicated by dotted lines extending from the rectangle to the outermost data points that were not considered outliers, and the outliers were plotted individually using the “+” symbol.

## Data Availability

The data that support the findings of this study are available from the corresponding author, M.M., upon reasonable request.

## Supplementary Materials

The supporting information can be downloaded at: https://www.mdpi.com/article/10.3390/ijms25126385/s1.
