# Supplementary material for: Dexmedetomidine as a Short-Use Analgesia for the Immature Nervous System
Source: Int J Mol Sci. 2024 Jun 9;25(12):6385. doi: 10.3390/ijms25126385 (PMC11204225; doi:10.3390/ijms25126385)
Supplement: Supplementary file 1 [file ijms-25-06385-s001.zip › ijms-2982492-supplementary.pdf]

Supplementary Materials:

Table S1. Duration of DEX effect (figures 1B, 2A, 4C)

	median	25%	75%
Contunuity of cortex activity	25.5	20.0	28.5
eSPW occurrence	23.5	21.0	29.0
Movements	20.0	16.0	25.0
Respiratory rate	26.0	23.5	30.0
Heart rate	23.0	18.5	26.5

Table S2. Continuity of cortical activity (figure 1C)

	absolute values			normalized values			p-value map		
	median	25%	75%	median	25%	75%	Control	Dex	Urethane
Control	0.3889	0.3119	0.4344	1	1	1	1	0.0002	0.0002
Dex	0.2264	0.1470	0.3154	0.6249	0.4004	0.8042	0.0002	1	0.0148
Urethane	0.0845	0.0648	0.1367	0.3002	0.1588	0.4162	0.0002	0.0148	1

Table S3. Integrative power of alpha-beta range (figure 1D)

	absolute values			normalized values			p-value map		
	median	25%	75%	median	25%	75%	Control	Dex	Urethane
Control	1318.2178	925.3015	1893.8234	1	1	1	1	0.4042	0.0002
Dex	1309.4263	781.5520	1872.3422	0.8642	0.7281	1.1136	0.4042	1	0.0002
Urethane	730.4083	447.4212	833.3661	0.5186	0.4016	0.5583	0.0002	0.0002	1

Table S4. Integrative power of gamma range (figure 1E)

	absolute values			normalized values			p-value map		
	median	25%	75%	median	25%	75%	Control	Dex	Urethane
Control	166.7148	117.6640	210.4702	1	1	1	1	0.0831	0.0002
Dex	129.0463	82.3872	209.2075	0.7548	0.6663	1.0019	0.0831	1	0.0047
Urethane	82.9959	61.7911	119.3629	0.5636	0.4882	0.6182	0.0002	0.0047	1

Table S5. eSPW occurrence (figure 2B)

	absolute values			normalized values			p-value map		
	median	25%	75%	median	25%	75%	Control	Dex	Urethane
Control	2.9292	1.8902	5.8163	1	1	1	1	0.0002	0.0002
Dex	1.7478	1.0825	2.8062	0.5426	0.4506	0.6220	0.0002	1	0.0047
Urethane	0.8189	0.6381	0.8785	0.2752	0.1553	0.4475	0.0002	0.0047	1

Table S6. eSPW duration (figure 2C)

	absolute values			normalized values			p-value map		
	median	25%	75%	median	25%	75%	Control	Dex	Urethane
Control	102.0353	93.4327	109.7268	1	1	1	1	0.4042	0.4042
Dex	106.9731	102.9226	114.0936	1.0414	0.9820	1.1450	0.4042	1	0.1049
Urethane	92.4107	85.0978	104.8402	0.9461	0.8403	1.0338	0.4042	0.1049	1

Table S7. eSPW amplitude in stratum oriens (figure 2D)

	absolute values			normalized values			p-value map		
	median	25%	75%	median	25%	75%	Control	Dex	Urethane
Control	48.4657	34.6005	76.9024	1	1	1	1	0.0831	0.0002
Dex	57.6080	35.2735	64.8641	1.2033	0.8524	1.2588	0.0831	1	0.0104
Urethane	18.3324	13.2507	38.3758	0.5683	0.2781	0.6352	0.0002	0.0104	1

Table S8. eSPW amplitude in stratum radiatum (figure 2E)

	absolute values			normalized values			p-value map		
	median	25%	75%	median	25%	75%	Control	Dex	Urethane
Control	-99.0797	-121.9958	-72.1352	1	1	1	1	0.0057	0.0057
Dex	-57.1095	-84.2135	-41.1632	0.7781	0.5072	0.9449	0.0057	1	0.5737
Urethane	-51.8005	-89.8811	-31.4673	0.6357	0.3993	0.9311	0.0057	0.5737	1

Table S9. Tail-Flick time (figure 3A)

	absolute values			normalized values			p-value map		
	median	25%	75%	median	25%	75%	Control	Dex	Urethane
Control	0.9150	0.7300	1.2800	1	1	1	1	0.0001	0.0001
Dex	1.6600	1.3840	1.9000	1.8797	1.4037	1.9141	0.0001	1	0.2413
Urethane	1.8775	1.6700	2.1000	2.1064	1.8621	2.5615	0.0001	0.2413	1

Table S10. Active sleep (figure 3D)

	absolute values			p-value map		
	median	25%	75%	Control	Dex	Urethane
Control	0.6310	0.5779	0.7157	1	0.4557	0.0006
Dex	0.5950	0.4137	0.6502	0.4557	1	0.0006
Urethane	0	0	0	0.0006	0.0006	1

Table S11. Quiet sleep (figure 3D)

	absolute values			p-value map		
	median	25%	75%	Control	Dex	Urethane
Control	0.1653	0.1440	0.2301	1	0.0379	0.0006
Dex	0.2771	0.2313	0.3815	0.0379	1	0.0006
Urethane	1	1	1	0.0006	0.0006	1

Table S12. Wake state (figure 3D)

	absolute values			p-value map		
	median	25%	75%	Control	Dex	Urethane
Control	0.2037	0.1067	0.2401	1	0.4557	0.0006
Dex	0.1790	0.0889	0.2270	0.4557	1	0.0006
Urethane	0	0	0	0.0006	0.0006	1

Table S13. Twitches occurrence (figure 3E)

	absolute values			normalized values			p-value map		
	median	25%	75%	median	25%	75%	Control	Dex	Urethane
Control	6.3623	5.9892	10.4601	1	1	1	1	0.0006	0.0006
Dex	3.9100	3.7671	4.3077	0.6146	0.4727	0.7071	0.0006	1	0.0006
Urethane	0.0727	0.0482	0.1427	0.0083	0.0066	0.0181	0.0006	0.0006	1

Table S14. Movements (figure 4B)

	absolute values			normalized values			p-value map		
	median	25%	75%	median	25%	75%	Control	Dex	Urethane
Control	0.2350	0.2213	0.3953	1	1	1	1	0.0002	0.0002
Dex	0.1133	0.0992	0.1660	0.4176	0.3851	0.6104	0.0002	1	0.0002
Urethane	0.0169	0.0080	0.0296	0.0398	0.0352	0.0973	0.0002	0.0002	1

Table S15. Respiratory rate (figure 4E)

	absolute values			normalized values			p-value map		
	median	25%	75%	median	25%	75%	Control	Dex	Urethane
Control	113.3333	103.6833	138.5917	1	1	1	1	0.0006	0.0006
Dex	72.5500	63.5125	77.9625	0.6104	0.5480	0.6386	0.0006	1	0.0012
Urethane	109.0000	69.8583	114.3250	0.8075	0.7522	0.8790	0.0006	0.0012	1

Table S16. Heart rate (figure 4F)

	absolute values			normalized values			p-value map		
	median	25%	75%	median	25%	75%	Control	Dex	Urethane
Control	514.5333	436.5475	535.9417	1	1	1	1	0.0006	0.0006
Dex	313.4500	258.9013	367.0850	0.6104	0.5424	0.6999	0.0006	1	0.0041
Urethane	441.5333	310.2450	450.7355	0.8167	0.7623	0.8926	0.0006	0.0041	1

Table S17. Parameters of cortical activity by separated urethane injections (figures 1C-E, blue filled circles)

Animals	Contunuity of cortical activity	Integrative power of alpha-beta range	Integrative power of gamma range
№1 (p6)	0.1227	628.9796	106.3075
№2 (p7)	0.0889	777.2814	138.8433

Table S18. Continuity of cortical activy (figure 1C, filled circles, 3 neonatal rats of P5-7)

	absolute values			normalized values		
	median	25%	75%	median	25%	75%
Control	0.4269	0.4128	0.4382	1	1	1
Sham	0.4347	0.4063	0.4554	1.0182	0.9839	1.0391
DEX (1.5 ug/kg)	0.4229	0.3766	0.4850	0.9569	0.9028	1.1277

Table S19. Integrative power of alpha-beta range (figure 1D, filled circles, 3 neonatal rats of P5-7)

	absolute values			normalized values		
	median	25%	75%	median	25%	75%
Control	965.9016	783.7535	1091.8741	1	1	1
Sham	1039.5143	771.2144	1124.4039	1.0166	0.9614	1.0613
DEX (1.5 ug/kg)	896.6895	754.7187	1308.7044	0.9784	0.9408	1.2011

Table S20. Integrative power of gamma range (figure 1E, filled circles, 3 neonatal rats of P5-7)

	absolute values			normalized values		
	median	25%	75%	median	25%	75%
Control	113.0608	97.2967	126.5515	1	1	1
Sham	118.4965	98.3203	135.4447	0.9951	0.9270	1.1847
DEX (1.5 ug/kg)	122.2378	95.0371	135.8774	0.9340	0.9331	1.1650

Table S21. eSPW occurrence (3 neonatal rats of P5-7)

	absolute values			normalized values		
	median	25%	75%	median	25%	75%
Control	1.7565	1.6851	2.2836	1	1	1
Sham	1.9361	1.7817	2.0164	1.0415	0.8508	1.1328
DEX (1.5 ug/kg)	1.8144	1.6832	1.8847	0.9869	0.8000	1.0615

Table S22. eSPW duration (3 neonatal rats of P5-7)

	absolute values			normalized values		
	median	25%	75%	median	25%	75%
Control	92.3061	88.0622	105.8301	1	1	1
Sham	97.8922	95.8612	100.6932	1.0605	0.9559	1.0890
DEX (1.5 ug/kg)	105.0857	92.5083	106.7893	1.0193	0.9691	1.1271

Table S23. eSPW amplitude in stratum oriens (3 neonatal rats of P5-7)

	absolute values			normalized values		
	median	25%	75%	median	25%	75%
Control	45.7178	29.8367	56.3596	1	1	1
Sham	36.6799	29.2160	51.8248	0.9494	0.8391	1.0541
DEX (1.5 ug/kg)	37.5988	27.1959	58.9193	0.9668	0.8585	1.0683

Table S24. eSPW amplitude in stratum radiatum (3 P5-7 neonatal rats)

	absolute values			normalized values		
	median	25%	75%	median	25%	75%
Control	-114.2861	-125.8507	-100.9704	1	1	1
Sham	-108.9288	-118.3364	-105.0690	0.9531	0.9407	1.0446
DEX (1.5 ug/kg)	-110.7797	-122.1919	-97.8087	0.9684	0.8827	1.0690

Table S25. Tail-Flick time (7 P6-7 neonatal rats)

	absolute values			normalized values		
	median	25%	75%	median	25%	75%
Control	0.7300	0.5475	0.8600	1	1	1
Sham	0.6500	0.6175	0.7525	0.9615	0.8712	1.0238
DEX (1.5 ug/kg)	0.6300	0.6100	0.7600	1.0115	0.7300	1.1435

Table S26. Active sleep (3 P5-7 neonatal rats)

	absolute values			normalized values		
	median	25%	75%	median	25%	75%
Control	0.6380	0.5954	0.7396	1	1	1
Sham	0.6427	0.6152	0.7145	1.0428	0.8840	1.1288
DEX (1.5 ug/kg)	0.6001	0.5334	0.7117	1.0326	0.7538	1.1384

Table S27. Quiet sleep (3 P5-7 neonatal rats)

	absolute values			normalized values		
	median	25%	75%	median	25%	75%
Control	0.1412	0.1368	0.2249	1	1	1
Sham	0.1981	0.1745	0.2138	1.4636	0.8600	1.5296
DEX (1.5 ug/kg)	0.2006	0.1524	0.3214	1.4827	0.7750	2.2921

Table S28. Wake state (3 P5-7 neonatal rats)

	absolute values			normalized values		
	median	25%	75%	median	25%	75%
Control	0.1091	0.0913	0.2399	1	1	1
Sham	0.1382	0.1058	0.1815	0.8702	0.7357	1.4313
DEX (1.5 ug/kg)	0.1273	0.1179	0.1812	1.0524	0.7902	1.3804

Table S29. Twitches occurrence (3 P5-7 neonatal rats)

	absolute values			normalized values		
	median	25%	75%	median	25%	75%
Control	11.0372	9.3058	11.6389	1	1	1
Sham	10.6178	10.4441	12.3076	1.0871	0.9775	1.1841
DEX (1.5 ug/kg)	9.9685	9.7977	11.3039	1.0645	0.8832	1.1227

Table S30. Movements (3 P5-7 neonatal rats)

	absolute values			normalized values		
	median	25%	75%	median	25%	75%
Control	0.2696	0.1749	0.4582	1	1	1
Sham	0.2637	0.1881	0.3767	0.9781	0.8410	1.0963
DEX (1.5 ug/kg)	0.2532	0.1788	0.3838	0.9393	0.8499	1.0401

Table S31. Respiratory rate (3 P5-7 neonatal rats)

	absolute values			normalized values		
	median	25%	75%	median	25%	75%
Control	107.7000	106.3000	134.6000	1	1	1
Sham	126.2667	114.3167	128.5667	1.0245	0.9318	1.1509
DEX (1.5 ug/kg)	115.1000	111.6125	122.9000	1.0255	0.9120	1.0721

Table S32. Heart rate (3 P5-7 neonatal rats)

	absolute values			normalized values		
	median	25%	75%	median	25%	75%
Control	558.4743	525.5186	582.8561	1	1	1
Sham	582.2333	519.7083	582.9333	0.9852	0.9162	1.0963
DEX (1.5 ug/kg)	588.5500	537.4375	588.6250	0.9961	0.9479	1.1069
